# Statistical-based optimization and scale-up of siderophore production process on laboratory bioreactor

**DOI:** 10.1007/s13205-016-0365-2

**Published:** 2016-02-15

**Authors:** S. S. Shaikh, S. J. Wani, R. Z. Sayyed

**Affiliations:** Department of Microbiology, PSGVP Mandal’s, Arts, Science and Commerce College, Shahada, Dist Nandurbar, Maharashtra 425 409 India

**Keywords:** Optimization, Plackett–Burman (PB) design, Response surface methodology (RSM), Scale up, Siderophore

## Abstract

We report the enhanced production of siderophore in succinate medium by applying two-stage statistical approach, i.e., Plackett–Burman design and response surface methodology (RSM) using central composite design (CCD). In the first stage of optimization, out of 11 variable components of succinate medium, succinic acid, pH and temperature were found as significant components that influenced the siderophore production in *Pseudomonas aeruginosa* RZS9. The second stage of RSM using CCD consisted of optimizing the concentrations of the variables. Here, 0.49 g/100 ml concentration of succinic acid, pH 7.08 and temperature of 27.80 °C yielded the maximum (68.41 %) siderophore units. All the significant components exhibited quadratic effect on siderophore production. The *F* value of 28.63, multiple correlation coefficient (*R* ^2^) of 0.9626, percent coefficient of variation of 8.81 values indicated that the model was significant and that the experimental data was satisfactorily adjusted to the quadratic model. During validation of these experiments, 6.10 % increase in siderophore yield was obtained. Scale-up of this protocol optimized at shake  flask level up to 5 L-capacity reactor further enhanced the siderophore yield. We claim it to be the first report on statistical optimization of siderophore production by *P. aeruginosa* RZS9.

**Electronic supplementary material:**

The online version of this article (doi:10.1007/s13205-016-0365-2) contains supplementary material, which is available to authorized users.

## Introduction

Siderophores are low-molecular-weight, iron-scavenging ligands produced by a wide variety of microorganisms to combat iron deficiency (Sayyed et al. 2013). Siderophores of rhizobacteria provide iron nutrition to the plants and help in plant-growth promotion. They prevent the plant pathogens from iron nutrition; thereby restricting its growth, and thus, help in biological control of phytopathogens (Shaikh et al. 2014). Besides these, siderophores are also used in pharmaceuticals (Crumbliss and Harrington 2009; Hider and Kong 2010), bioremediation of heavy metal pollutants (Rajkumar et al. 2010), biogeochemical cycling of iron in the ocean (Boyd et al. 2007), biodegradation of petroleum hydrocarbons (Hickford et al. 2004; Gauglitz et al. 2012), and as an optical biosensor (Yoder and Kisaalita 2011; Ahmed and Holmström 2014). Since these molecules have wider range of applications, their production needs to be statistically optimized. Hence, any factor, which influences the production of siderophores in a production medium, needs to be studied.

Non-statistical optimization approach considers only one factor at a time. It is tedious and time-consuming, especially for multi-variable screening, and it does not consider the complex interactions among different variables (Bas and Boyac 2007; Hegde et al. 2013). Statistical-based approaches offer ideal ways for process-optimization studies in several biochemical and biotechnological processes (Bas and Boyac 2007) such as amylase production (Gangadharan et al. 2008; Prajapati et al. 2015), ethanol production (Mei et al. 2009), hydrogen production (Guo et al. 2009), phytase production (Singh and Satyanarayana 2008), avermectin production (Gao et al. 2009), phenazine-1-carboxylic acid production (Su et al. 2010), cellulose production (Mohite et al. 2012), and cellulase production (Hegde et al. 2013; Thakkar and Saraf 2014). However, there are very few reports on statistical optimization of siderophore production.

Present work focuses on statistical-based optimization approaches, i.e., Plackett–Burman (PB) design and response surface methodology (RSM) by central composite design (CCD), for enhanced production of siderophore by using succinate medium. Scale-up of optimized shake flask protocol to laboratory-scale bioreactor was also studied.

## Materials and methods

### Source of culture

The siderophore-producing bacterial isolate used in this experiment was isolated and identified as *Pseudomonas aeruginosa* on the basis of biochemical characteristics, 16s rRNA sequencing, Biolog and GC-FAME analysis (Data not shown). The culture was submitted to the gene bank as *P. aeruginosa* RZS9 under National Center for Biotechnology information (NCBI) accession number KP866815.

### Production, detection and quantification of siderophore

Production of siderophore was carried out in erlenmeyer flask containing 100 ml succinate medium (Meyer and Abdallah 1978). For this purpose, *P. aeruginosa* RZS9 (6 × 10^6^ cells ml^−1^ corresponding to 0.20 OD units) was grown in succinate medium at 28 ± 2 °C at 120 rpm for 24 h, followed by measuring cell density at 620 nm by using double-beam UV–visible spectrophotometer (1240, Shimadzu, Japan). The detection and estimation of siderophore was performed after centrifugation (15 min at 5000*g* × cm at 4 °C), and the cell-free supernatant was tested for the presence of siderophore by using CAS test (Schwyn and Neilands 1987). CAS shuttle assay was used for quantitative estimation of siderophore (Payne 1994); in this, 1 ml of the culture supernatant was mixed with 1 ml of CAS reagent. Absorbance was measured at 630 nm against a reference consisting of 1 ml of uninoculated broth and 1 ml of CAS reagent. Siderophore content in the aliquot was calculated by using the following formula and expressed as percent siderophore unit (SU), which is defined as the percent (v/v) of siderophore present in the given sample:

$$\% {\text{ SU}} = \frac{{{\text{Ar}} - {\text{As}}}}{\text{Ar}} \times 100$$

where Ar = Absorbance of the reference at 630 nm (CAS reagent), As = Absorbance of the sample at 630 nm.

### Designs of experiment

In the first phase, PB design was used to find the significant variable(s) or factor(s) to optimize the siderophore production. In the next phase, RSM through CCD was used to find the optimum concentration of the selected variable. The effect of selected variables on the responses was analyzed to maximize the siderophore production (Murugappan et al. 2007).

### Plackett–Burman design

In the first phase of the optimization, 11 variable PB experimental design was used to find the significant ingredients of the medium for the maximum production of siderophore (Plackett and Burman 1946). This experimental design was two-factorial design, and was used to identify the critical parameters required for optimum siderophore production by screening n variables in n + 1 experiment (Plackett and Burman 1946). The components of succinic acid medium chosen for the present study were K_2_HPO_4_, KH_2_PO_4_, (NH_4_) SO_4_, MgSO_4_·7H_2_O and succinic acid. Physical parameters such as pH and temperature also influence the siderophore production. These variables were coded as A, B, C, D, E, F and G respectively, while H, I, J, and K were considered as dummy variables. All the independent variables with their H and L value are described in Table 1.

**Table 1:** Medium components and their variables used in Placket–Burman design for siderophore production

Variable code	Variable	High value (+1)	Low value (−1)
*A*	K_2_HPO_4_	0.78	0.42
*B*	KH_2_PO_4_	0.39	0.21
*C*	(NH_4_)SO_4_	0.13	0.07
*D*	MgSO_4_·7H_2_O	0.026	0.014
*E*	Succinic acid	0.52	0.28
*F*	pH	6	8
*G*	Temperature	22	34

The experimental design for the screening of these variables is presented in Table 2. Experiments of PB design were done in triplicates, and the average mean of SU was considered as the final response. The quantification of siderophore production was done by CAS shuttle assay (Payne 1994). Each of these variables was represented at two different levels—high concentration (H) and low concentration (L). The effect of individual variable was determined by calculating the difference between the average of measure at the H value and the L value. The full experimental design consisting of 11 variables with four dummy variables and 12 trials is depicted in Table 2. The effect of each variable on siderophore production in the form of SU was calculated, and the highest confidence level and *f* test was used to determine the significant component (Stanbury et al. 2003).

**Table 2:** 11 variable Plackett–Burman experimental design

Run	*A*	*B*	*C*	*D*	*E*	*F*	*G*	*H*	*I*	*J*	*K*	Yield % SU
1	H	H	H	H	H	H	H	H	H	H	H	50.94
2	L	H	L	H	H	H	L	L	L	H	L	52.31
3	L	L	H	L	H	H	H	L	L	L	H	50.14
4	H	L	L	H	L	H	H	H	L	L	L	45.84
5	L	H	L	L	H	L	H	H	H	L	L	45.35
6	L	L	H	L	L	H	L	H	H	H	L	48.36
7	L	L	L	H	L	L	H	L	H	H	H	40.65
8	H	L	L	L	H	L	L	H	L	H	H	49.35
9	H	H	L	L	L	H	L	L	H	L	H	48.01
10	H	H	H	L	L	L	H	L	L	H	L	40.86
11	L	H	H	H	L	L	L	H	L	L	H	44.75
12	H	L	H	H	H	L	L	L	H	L	L	50.32

### Response surface methodology (RSM)

The second phase in optimization of the medium-component was to find the optimum concentration of the significant components by RSM through CCD. It involved steps such as procedures to find the optimum region, the responses in the optimum region of variables, estimation of the optimal conditions and verification of the data (Tanyildizi et al. 2005). The variables obtained from PB design to enhance the siderophore production were selected for CCD to study the interaction between the various medium constituents, which influence the siderophore production. The CCD was used to study the interaction between the significant components and also to determine their optimum levels. In the present work, experiments were planned to obtain a quadratic model. Hence, the concentrations of the three factors i.e., succinic acid, pH and temperature, identified by PB design, were optimized, keeping other variables constant at zero (0) level. Each factor was studied at five different levels (−*α*, −1, 0, +1, +*α*) (Table 3). The complete experimental plan of CCD with respect to their values in coded and actual form is listed in Table 4. SUs were measured in triplicate in 20 trial-experimental runs. Design matrix consists of coded terms with eight factorial points, six axial points and five central points. The data were fitted into the second-order polynomial equation, and the coefficients were calculated and analysed. The general form of the second-degree polynomial equation is:

$$Yi \, = \, \beta 0 \, + \, \sum \, \beta iXi \, + \, \sum \beta iiXi^{2} + \, \sum \beta ijXiXj$$

where *Yi* is the predicted response, *XiXj* are input variables, which influence the response variable *Y*, *β*0 is the offset term, *βi* is the *i*th linear coefficient, *βii* is the quadratic coefficient, *βij* is the *ij*th interaction coefficient.

**Table 3:** Experimental range and levels of the independent variables components used for response surface central composite design

Variable	Components	−*α*	−1	0	+1	+*α*
*A*	Succinic acid	0.198185	0.28	0.4	0.52	0.601815
*B*	pH	5.31821	6	7	8	8.68179
*C*	Temperature	17.9092	22	28	34	38.0908

**Table 4:** Analysis of siderophore yield by Plackett–Burman experimental design

	Difference	Effect	Mean square	*t* value	*P* value	*F* value	Confidence level
*A*	3.76	0.94	1.7672	4.88819553	0.00811258	5.7941	99.18
*B*	−2.44	−0.61	0.7442	−3.17212689	0.03378888	2.44	96.62
*C*	3.86	0.965	1.86245	5.01820073	0.00739474	6.10639	99.26
*D*	2.74	0.685	0.93845	3.56214249	0.02354079	3.07689	97.64
*E*	29.94	7.485	112.05	38.9235569	2.6025E−06	367.379	99.99
*F*	24.32	6.08	73.9328	31.6172647	5.9644E−06	242.403	99.99
*G*	−19.32	−4.83	46.6578	−25.1170047	1.4918E−05	152.976	99.99
*H*	2.3	0.575	0.66125	2.9901196	ND	ND	ND
*I*	0.38	0.095	0.01805	0.49401976	ND	ND	ND
*J*	−1.94	−0.485	0.47045	−2.52210088	ND	ND	ND
*K*	0.8	0.2	0.08	1.0400416	ND	ND	ND

Standard error 0.1923, Mean square for error 0.305, *ND* not determined

After the analysis of data, the experimental run with optimum values of variable was done to check the validity of the model.

### Scale-up on bioreactor

The shake flask process was scaled-up to 5L capacity fully-automated laboratory-scale bioreactor [Murhopye Scientific Co., India, Model LF-5] to check the performance of siderophore producing isolate and to confirm the validity of optimization studies carried out at shake flask level.

### Software and data analysis

Analysis and interpretation of the data was carried out for the RSM experimental design using Design-Expert version 9.0.3.1 (Stat-Ease Inc., Minneapolis, MN, USA) statistical software.

## Results and discussion

### Production, detection and quantification of siderophore

After 24 h incubation of inoculated succinate medium, change in the colour of the medium from colourless to fluorescent-green indicated siderophore-producing ability of *P. aeruginosa* RZS9. Positive CAS test, i.e., change in the colour of CAS from blue to orange-yellow confirmed the siderophore production. *P. aeruginosa* RZS9 produced 63.38 % SU. Similar results have been reported by Milagres et al. (1999), Sayyed and Patel (2011), and Shaikh et al. (2014).

### Plackett–Burman design

The medium components are known to have varying effect on the siderophore production. PB design was used to screen seven different medium-components in 12 trial-experimental runs with two levels, high (H) and low (L) of each variable. The independent variables and their respective H and L value used in the optimization study, and the response in the form of SU are represented in Table 3, whereas the analysis of the yield by PB experimental design are represented in Table 4, in which the variables showing higher *F* value are considered as significant and those showing *F* value near zero (0) are considered as insignificant (Stanbury et al. 2003). Level of significance was also determined by highest confidence level. Hence, succinic acid, pH and temperature were considered as significant components in siderophore production.

### Response surface methodology (RSM)

To study the combined effect of these three variables, Trials were performed at different combinations of each variable to study the combined effect of these three variables. The CCD experimental plan along with experimental response and the predicted response for each individual experiment is scrutinized in Table 5. It shows the production of siderophore corresponding to the combined effect of all three components in the specific range. The production of siderophore may be predicted by the equation derived by Design-Expert software. This final equation, in terms of Coded Factors, can be used to make predictions about the response for given levels of each factor. Here, the levels should be specified in the original units for each factor.

$$\begin{aligned} Y \, & = + 63.94 \, + 10.99\left( A \right) \, + 3.14 \, \left( B \right) \, - 0.34 \, \left( C \right) \, - 1.37 \, \left( {AB} \right) \, - 0.19\left( {AC} \right) \, \\ & \quad + 1.12\left( {BC} \right) \, - 6.76\left( A \right)^{2} - 11.84 \, \left( B \right)^{2} - 5.75 \, \left( C \right)^{2} \\ \end{aligned}$$

where, *Y* = SU in  %, *A* = succinic acid, *B* = pH, *C* = temperature.

**Table 5:** Full experimental central composite design with coded and actual level of variables and the response function

Run	Type	A:Succinic acid (g/100 ml)		B:pH		C:Temperature (°C)		Siderophore unit %	
		Coded	Actual	Coded	Actual	Coded	Actual	Experimental	Predicted
1	Factorial	+1	0.52	−1	6	−1	22	44.67	50.02
2	Factorial	+1	0.52	+1	8	+1	34	48.87	52.49
3	Factorial	−1	0.28	+1	8	−1	22	29.58	32.26
4	Axial	+*α*	0.601815	0	7	0	28	70.97	61.89
5	Axial	0	0.4	−*α*	5.31821	0	28	26.46	25.42
6	Center	0	0.4	0	7	0	28	63.97	63.93
7	Factorial	0	0.52	+1	8	−1	22	46.86	51.32
8	Axial	−*α*	0.198185	0	7	0	28	23.76	25.89
9	Factorial	−1	0.28	−1	6	−1	22	25.84	25.49
10	Factorial	−1	0.28	+1	8	+1	34	36.28	34.21
11	Center	0	0.4	0	7	0	28	63.73	63.93
12	Axial	0	0.4	0	7	+*α*	38.0908	46.34	47.35
13	Factorial	−1	0.28	−1	6	+1	34	24.15	22.96
14	Axial	0	0.4	+*α*	8.68179	0	28	39.56	35.97
15	Center	0	0.4	0	7	0	28	63.27	63.93
16	Factorial	+1	0.52	−1	6	+1	34	46.13	46.72
17	Center	0	0.4	0	7	0	28	63.42	63.93
18	Center	0	0.4	0	7	0	28	64.32	63.93
19	Axial	0	0.4	0	7	−*α*	17.9092	54.13	48.49
20	Center	0	0.4	0	7	0	28	64.08	63.93

The statistical significance of the second-order quadratic model was evaluated by *F* test ANOVA, which revealed that this regression model is at a statistically-higher significance level for siderophore production. The *F* value of the model was 28.63, which implies that the model is statistically significant. There is only 0.01 % chance that an *F* value of this large could occur due to noise. Values of ‘Prob > *F*’ less than 0.0500 indicated that the model is significant (Table 6). The large ‘Lack of Fit *F* value’ also indicated that the Lack of Fit is significant. Multiple correlation coefficient (*R* ^2^) reflected the adequacy of this model. The *R* ^2^ value of 0.9626 closer to 1 denoted better correlation between predicted and observed values (Gao et al. 2009; Mohite et al. 2012; Thakkar and Saraf 2014). It indicated that the model could explain 96 % of the variability. The coefficient of variation (CV) indicates the degree of precision in which the experiments were compared. The low reliability of the model-experiments was indicated by the high value of CV. In the present case, a low CV (8.81) denoted that the experiments performed are reliable (Gangadharan et al. 2008; Prajapati et al. 2015). ‘Adeq Precision’ measures the signal to noise ratio. A ratio >4 is desirable. Here, the value of 13.932 indicates an adequate signal. Thus, this model can be used to navigate the design space, and the model is best fitted for the optimization studies (Prajapati et al. 2015).

**Table 6:** Analysis of variance of quadratic model for siderophore production

Source	Squares	*df*	Square	Value	Prob > *F*
Model	4455.44	9	495.05	28.63	<0.0001 significant
*A*-succinic acid	1564.59	1	1564.59	90.48	<0.0001
*B*-pH	134.33	1	134.33	7.77	0.0192
*C*-temp	1.56	1	1.56	0.090	0.7698
*AB*	14.96	1	14.96	0.87	0.3742
*AC*	0.30	1	0.30	0.017	0.8984
*BC*	9.99	1	9.99	0.58	0.4647
*A* ^2^	723.48	1	723.48	41.84	<0.0001
*B* ^2^	1989.83	1	1989.83	115.07	<0.0001
*C* ^2^	461.76	1	461.76	26.70	0.0004
Residual	172.93	10	17.29		
Lack of fit	172.12	5	34.42	213.05	<0.0001 significant
Pure error	0.81	5	0.16		
Cor total	4628.37	19			

*R* ^2^ = 0.9626, Standerd deviation = 4.16, CV% = 8.81, Adeq precision = 13.932

Pred *R* ^2^ = 0.7152, Adj *R* ^2^ = 0.9290

**Fig. 1:**
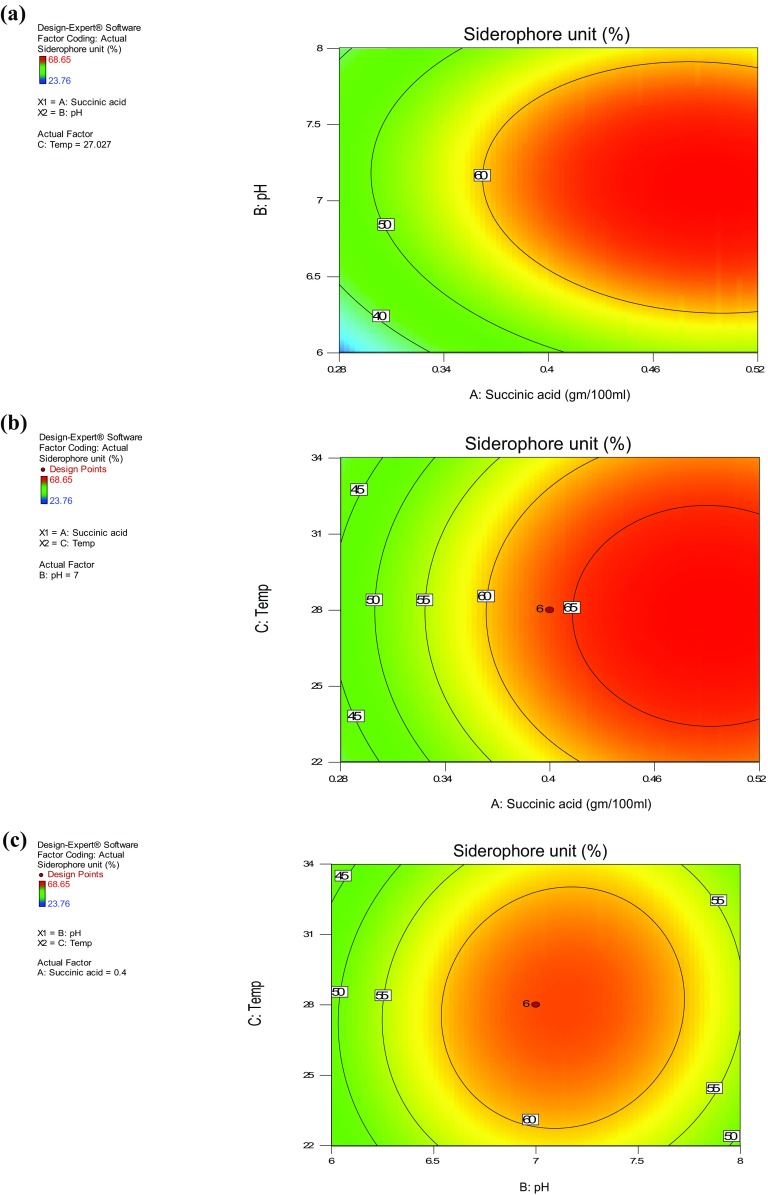
Counter plots showing interaction of different variables on siderophore production

**Fig. 2:**
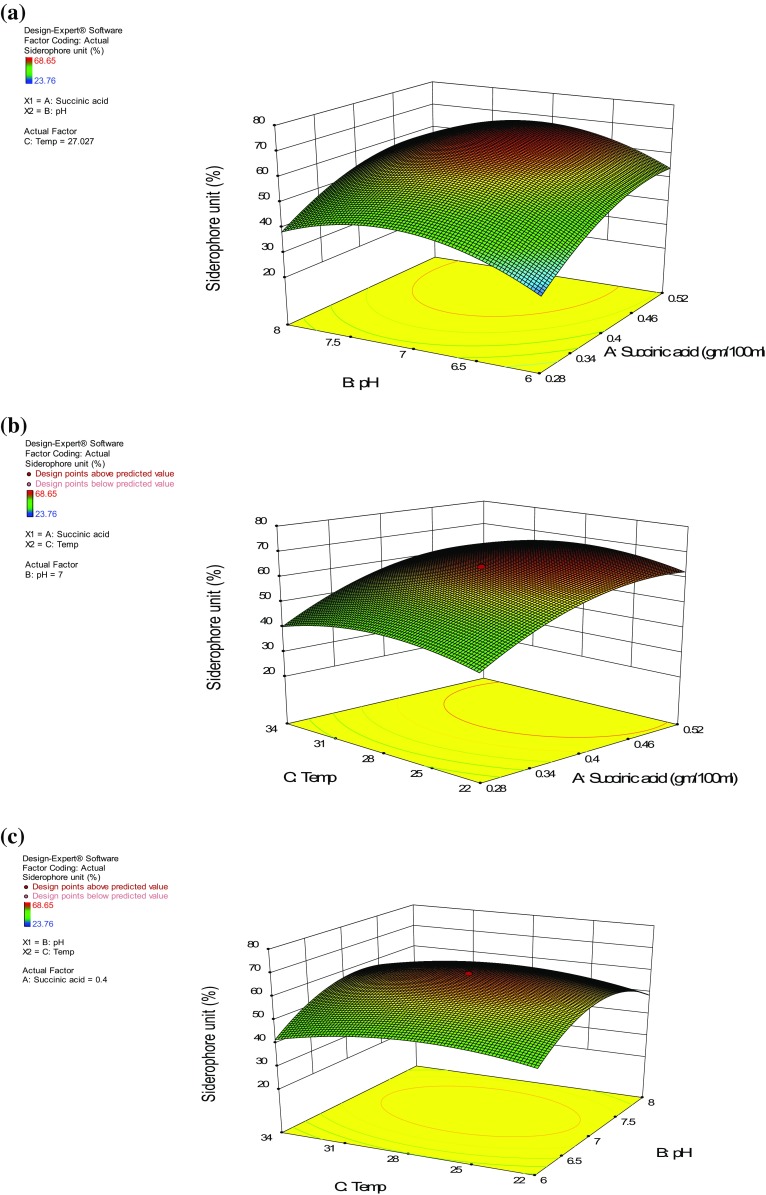
3D response surface plot showing interaction of different variables on siderophore production

**Fig. 3:**
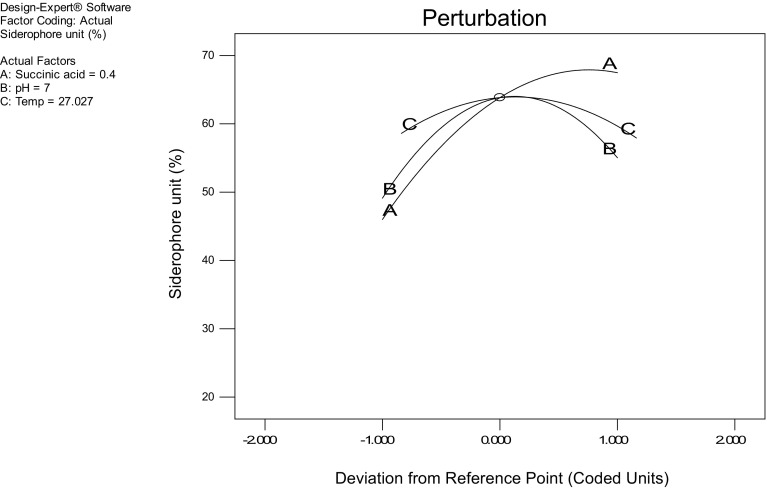
Pertubation plot for siderophore production as function of ammonium sulphate and succinic acid

The effect of variables on siderophore production was studied against three independent variables while maintaining the other independent variables at their zero (0) level. The three dimensional (3D) response surface plots were drawn to understand the interaction between medium components, and the optimum concentration of each variable required for maximum siderophore production. These contour plots (Fig. 1a–c) or 3D response surface plots (Fig. 2a–c) can be used to predict the optimal values for various test variables. Contour plots indicate the area of operability within the experimental region and helps to characterize the responses. These plots were obtained by considering all the possible combinations of all the variables under test. As observed in the 3D response surface plots, the shape of the curve shows a moderate interaction between any two variables when the third variable is constant.

**Fig. 4:**
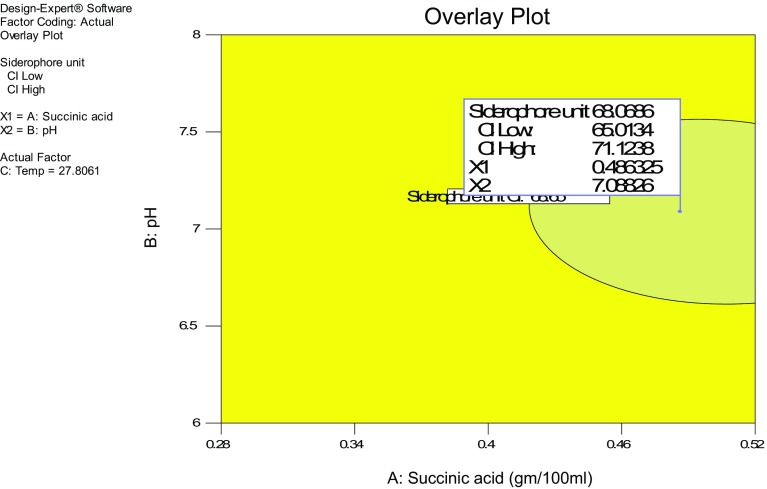
Overlay plot for siderophore production as function of ammonium sulphate and succinic acid

The effect of all the three factors on siderophore production as studied by perturbation plot in which comparison of the effect of all the factors at the zero (0) values (midpoint) in the design space was carried out, yielded a steep curvature or slope indicating the sensitivity of response to these factors (Fig. 3). As shown in Fig. 4, the concentration of succinic acid (X1) 0.49 g/100 ml at pH (X2) 7.08 and temperature of 27.80 °C yielded maximum (68.41 %) SU.

Validation of the experiment was carried out under the conditions predicted by the statistical model. The optimum concentrations estimated for the concentration of succinic acid (X1) 0.49 g/100 ml at pH (X2) 7.08 at a constant value of temperature 27.80 °C, yielded the maximum siderophore production of 68.41 %. Additional experiments in triplicate were carried out with the above-mentioned optimized medium to validate the predictions of the model. These experiments yielded the maximum siderophore production of 69.03 %. Hence, it is proved that the experimental results verified the validity predicted by the Design-Expert and the experimental results also confirm the optimal points.

### Scale-up on bioreactor

Scale-up of the shake flask optimized protocol to 5L capacity reactor resulted in further increase in siderophore yield by 1 %. 69.48 % siderophore yield was obtained after 24 h incubation in bioreactor. This confirmed the validity of optimization studies carried out at shake flask level.

## Conclusion

The statistical-based optimization offered an efficient and feasible approach. A 6.10 % increase in siderophore production was achieved with the optimized factors—concentration of succinic acid (X1) 0.49 g/100 ml at pH (X2) 7.08 at constant value of temperature 27.80 °C. This set up yielded the maximum siderophore production of 68.41 % vis-à-vis 63.58 % SU obtained in unoptimized protocol. Further increase in siderophore yield by 1 %, validated the success of scale-up studies to laboratory-scale bioreactor. The experimental values agreed with the predicted values generated by Design-Expert software.

## Electronic supplementary material

Below is the link to the electronic supplementary material.

Supplementary material 1 (PDF 105 kb)
