# Supplementary material for: Statistical-based optimization and scale-up of siderophore production process on laboratory bioreactor
Source: 3 Biotech. 2016 Feb 15;6(1):69. doi: 10.1007/s13205-016-0365-2 (PMC4754294; doi:10.1007/s13205-016-0365-2)
Supplement: Supplementary file 1 — Supplementary material 1 (PDF 105 kb) [file 13205_2016_365_MOESM1_ESM.pdf]

Nucleotide ▾

GenBank

Pseudomonas aeruginosa strain RZS9 16S ribosomal RNA gene, partial sequence

GenBank: KP866815.2

FASTA Graphics

Go to:

```
LOCUS       KP866815                1491 bp    DNA     linear   BCT 19-AUG-2015
DEFINITION  Pseudomonas aeruginosa strain RZS9 16S ribosomal RNA gene, partial
            sequence.
ACCESSION   KP866815
VERSION     KP866815.2  GI:918629571
KEYWORDS    .
SOURCE      Pseudomonas aeruginosa
  ORGANISM  Pseudomonas aeruginosa
            Bacteria; Proteobacteria; Gammaproteobacteria; Pseudomonadales;
            Pseudomonadaceae; Pseudomonas.
REFERENCE   1  (bases 1 to 1491)
  AUTHORS   Shaikh,S. and Sayyed,R.
  TITLE     Direct Submission
  JOURNAL   Submitted (28-FEB-2015) Microbiology, PSGVP Mandals ASC College
            Shahda, Shahada 425409, India
REFERENCE   2  (bases 1 to 1491)
  AUTHORS   Shaikh,S. and Sayyed,R.
  TITLE     Direct Submission
  JOURNAL   Submitted (19-AUG-2015) Microbiology, PSGVP Mandals ASC College
            Shahda, Shahada 425409, India
  REMARK    Sequence update by submitter
COMMENT     On Aug 19, 2015 this sequence version replaced gi:807068289.

            ##Assembly-Data-START##
            Sequencing Technology :: Sanger dideoxy sequencing
            ##Assembly-Data-END##
FEATURES             Location/Qualifiers
     source          1..1491
                     /organism="Pseudomonas aeruginosa"
                     /mol_type="genomic DNA"
                     /strain="RZS9"
                     /isolation_source="Rhizosphric soil"
                     /db_xref="taxon:287"
                     /country="India"
                     /collection_date="02-Nov-2012"
     rRNA            <1..>1491
                     /product="16S ribosomal RNA"
ORIGIN
        1 tctggctcag attgaacgct ggcggcaggc ctaacacatg caagtcgagc ggatgaaggg
       61 agcttgctcc tggattcagc ggcggacggg tgagtaatgc ctaggaatct gcctggtagt
      121 gggggataac gtccggaaac gggcgctaat accgcatacg tcctgaggga gaaagtgggg
      181 gatcttcgga cctcacgcta tcagatgagc ctaggtcgga ttagctagtt ggtggggtaa
      241 aggcctacca aggcgacgat ccgtaactgg tctgagagga tgatcagtca cactggaact
      301 gagacacggt ccagactcct acgggaggca gcagtgggga atattggaca atgggcgaaa
      361 gcctgatcca gccatgccgc gtgtgtgaag aaggtcttcg gattgtaaag cactttaagt
      421 tgggaggaag ggcagtaagt taataccttg ctgttttgac gttaccaaca gaataagcac
      481 cggctaactt cgtgccagca gccgcggtaa tacgaagggt gcaagcgtta atcggaatta
      541 ctgggcgtaa agcgcgcgta ggtggttcag caagttggat gtgaaatccc cgggctcaac
      601 ctgggaactg catccaaaac tactgagcta gagtacggta gagggtggtg gaatttcctg
      661 tgtagcggtg aaatgcgtag atataggaag gaacaccagt ggcgaaggcg accacctgga
      721 ctgatactga cactgaggtg cgaaagcgtg gggagcaaac aggattagat accctggtag
      781 tccacgccgt aaacgatgtc gactagccgt tgggatcctt gagatcttag tggcgcagct
      841 aacgcgataa gtcgaccgcc tggggagtac ggccgcaagg ttaaaactca aatgaattga
      901 cgggggcccg cacaagcggt ggagcatgtg gtttaattcg aagcaacgcg aagaacctta
      961 cctggccttg acatgctgag aactttccag agatggattg gtgccttcgg gaactcagac
     1021 acaggtgctg catggctgtc gtcagctcgt gtcgtgagat gttgggttaa gtcccgtaac
     1081 gagcgcaacc cttgtcctta gttaccagca cctcgggtgg gcactctaag gagactgccg
     1141 gtgacaaacc ggaggaaggt ggggatgacg tcaagtcatc atggccctta cggccagggc
     1201 tacacacgtg ctacaatggt cggtacaaag ggttgccaag ccgcgaggtg gagctaatcc
     1261 cataaaaccg atcgtagtcc ggatcgcagt ctgcaactcg actgcgtgaa gtcggaatcg
     1321 ctagtaatcg tgaatcagaa tgtcacggtg aatacgttcc cgggccttgt acacaccgcc
     1381 cgtcacacca tgggagtggg ttgctccaga agtagctagt ctaaccgcaa gggggacggt
     1441 taccacggag tgattcatga ctggggtgaa gtcgtaacaa ggtaaccgt a
//
```
